# The geometry of synchronization: quantifying the coupling direction of physiological signals of stress between individuals using inter-system recurrence networks

**DOI:** 10.3389/fnetp.2023.1289983

**Published:** 2023-11-01

**Authors:** Fred Hasselman, Luciënne den Uil, Renske Koordeman, Peter de Looff, Roy Otten

**Affiliations:** ^1^ Behavioural Science Institute, Radboud University, Nijmegen, Netherlands; ^2^ Department of Research and Development, Pluryn, Nijmegen, Netherlands; ^3^ Fivoor Science and Treatment Innovation, Den Dolder, Netherlands; ^4^ Department of Developmental Psychology, Tilburg University, Tilburg, Netherlands; ^5^ Specialized Forensic Care, De Borg National Expert Center, Den Dolder, Netherlands

**Keywords:** synchronization, complex networks, inter-system recurrence networks, coupling direction, physiological stress, severe intellectual disabilities

## Abstract

In the study of synchronization dynamics between interacting systems, several techniques are available to estimate coupling strength and coupling direction. Currently, there is no general ‘best’ method that will perform well in most contexts. Inter-system recurrence networks (IRN) combine auto-recurrence and cross-recurrence matrices to create a graph that represents interacting networks. The method is appealing because it is based on cross-recurrence quantification analysis, a well-developed method for studying synchronization between 2 systems, which can be expanded in the IRN framework to include N > 2 interacting networks. In this study we examine whether IRN can be used to analyze coupling dynamics between physiological variables (acceleration, blood volume pressure, electrodermal activity, heart rate and skin temperature) observed in a client in residential care with severe to profound intellectual disabilities (SPID) and their professional caregiver. Based on the cross-clustering coefficients of the IRN conclusions about the coupling direction (client or caregiver drives the interaction) can be drawn, however, deciding between bi-directional coupling or no coupling remains a challenge. Constructing the full IRN, based on the multivariate time series of five coupled processes, reveals the existence of potential feedback loops. Further study is needed to be able to determine dynamics of coupling between the different layers.

## 1 Introduction

In studies of the synchronization dynamics of interacting systems, many techniques are available to determine whether there is a (statistical) dependence between observed time series of the variables of interest, such as the cross-correlation function or the mutual information measure. A more complicated research question concerns causal inference from observed time series data, in which the goal is to quantify and qualify the nature of the coupling dynamics between the interacting systems in terms of strength and direction (unidirectional, symmetric, asymmetric bidirectional, etc.). Many different techniques are available like linear and nonlinear Granger causality (Granger, 1969; Marinazzo et al., 2011), various information theoretic measures such as transfer entropy (Schreiber, 2000), conditional mutual information (Paluš et al., 2001) and synergistic information (Quax et al., 2017), Cross-RQA (Zbilut et al., 1998; Shockley et al., 2002; Richardson et al., 2007) and Cross-Mapping techniques (Sugihara et al., 2012; Ma et al., 2014; Ma et al., 2017; Leng et al., 2020). The accurate identification of the coupling direction, however, remains a challenge (Feldhoff et al., 2012; Runge et al., 2019). Determining which technique is ‘best’ may depend on the specific context, because different techniques come with different sets of assumptions about the data generating process (e.g., linear, stationary, etc.) and different requirements for the observed data (e.g., time series length, noise levels). In addition, some measures of coupling direction have been shown to give inaccurate results based on the coupling strength and parameters such as frequency and phase differences between the signals (Feldhoff et al., 2012).

In the cognitive, behavioral and neurosciences, Cross-Recurrence Quantification analysis (CRQA) has frequently been used to study coupling dynamics of human behavior and physiology, for example, in postural sway, heart rate variability, interpersonal behavior and emotion dynamics (Shockley, 2005; Konvalinka et al., 2011; Cox et al., 2016; Main et al., 2016; Vink et al., 2018). In the present paper we demonstrate how measures obtained from inter-system recurrence networks (Feldhoff et al., 2012) can be used to analyze the synchronization between physiological signals that were simultaneously observed in two interacting individuals. Inter system recurrence networks are an extension of multilayer recurrence networks, in which the cross-recurrence matrix is used to quantify the interdependence between different network layers (Zou et al., 2019). The goal is not to present an optimal method for detecting coupling direction, but to explore the applicability of inter-system recurrence networks to analyze empirical data observed in a healthcare setting. One reason to choose the (multiplex) recurrence network framework is the potential for application in clinical practice (Hasselman, 2022). Due to affordable wearable sensors and electronic devices that allow experience sampling and momentary ecological assessment (self-reports of physiological, psychological, or emotional states), many healthcare institutes are looking for ways to integrate such process monitoring data into diagnostic and intervention (Fartacek et al., 2016; Olthof et al., 2019; Hasselman, 2022), and explore opportunities to personalize such procedures (Schiepek et al., 2016; Olthof et al., 2023).

The data analyzed in the present paper concern a client in residential care with severe to profound intellectual disabilities (SPID) and their professional caregiver, both equipped with wearable sensors which recorded a number of physiological signals (acceleration, blood volume pressure, electrodermal activity, heart rate and skin temperature) during regular daily shifts (Simons et al., 2021). A substantial percentage of people with SPID show severely challenging behavior, which most frequently includes aggression and self-injury (Janssen et al., 2002). In residential care, incidents in which SPID clients display challenging behavior are obviously a stressor for the client, but also for their environment, in particular the professional caregiver tasked with resolving as well as preventing such incidents. The frequency of occurrence and the severity of such incidents is known to affect caregivers’ wellbeing and consequently the quality of the care they are able to provide (Devereux et al., 2009). The focus of the analysis will be to assess the coupling strength and direction of physiological signals in the period before an incident with challenging behavior occurred (see Figure 4 for an example of the data). The goal is to gain insight in the role of physiological coupling dynamics between individuals leading up to an incident which may inform methods to reduce or prevent the frequency of occurrence and severity of such incidents. First, we formally introduce the concept of inter-system networks (IRN) and the measures that can be used to quantify coupling dynamics, second, we use simulated data to demonstrate the behavior of IRN measures in different coupling scenarios, finally we present an analysis of the empirical client-caregiver data using the same measures.

## 2 Inter-system recurrence networks (IRN)

Recurrence networks represent a graph-theoretical approach to time series analysis that is based on recurrence analysis, which evaluates the proximity of phase space trajectories. The structural properties of the recurrence network are associated to the geometry of the underlying attractor (Zou et al., 2019). In this section we describe how to construct a so-called inter-system recurrence network which is a representation of 2 (or more) interacting recurrence networks and examines to what extent the different systems can be understood to share the same state space or show evidence of coupled dynamics.

### 2.1 Constructing a recurrence network

An M dimensional multivariate timeseries ${\left\{y_{i}\right\}}_{i=1}^{N_{y}}$ with $y_{i}=y\left(t_{i}\right)$ that captures the relevant degrees of freedom of a complex system Y can be interpreted to represent its state evolution in an M dimensional state space. Each state is represented by an *m*-tuple of coordinates, the state vector $\vec{y_{i}}$ describes a trajectory through the M dimensional state space. To study the dynamics of system Y , first, a distance matrix is created that encodes the closeness of each observed state relative to all other states. Second, the selection of a distance threshold ε determines which states should be considered close, or recurring states. Applying the threshold to the distance matrix results in a binary *auto-recurrence matrix*

$$R_{ij}\left(\varepsilon \right)=R^{Y}\left(y_{i},y_{j}|\varepsilon \right)=\Theta \left(\varepsilon -\left\|\vec{y_{i}}-\vec{y_{j}}\right\|\right),\;i,j=1,\ldots ,N$$ (1)

in which $\left\|∙\right\|$ is a distance norm (e.g., Euclidean, Chebyshev) and $\Theta \left(∙\right)$ the Heaviside function, returning a 1 if a distance value is below the threshold ε , and 0 otherwise. The threshold ε determines how many recurrent states are found and is therefore monotonically related to the Recurrence Rate (RR), the number of ε -recurrent points on the maximal possible number of points. Often, a predetermined recurrence rate is used to select a threshold ε (e.g., 1%–5%). The recurrence matrix is used in Recurrence Quantification Analysis (RQA) to obtain estimates of dynamical invariants and complexity measures from the (distribution of) ε− recurrent points and the vertical and diagonal line structures they form (Marwan et al., 2007).

**FIGURE 1:**
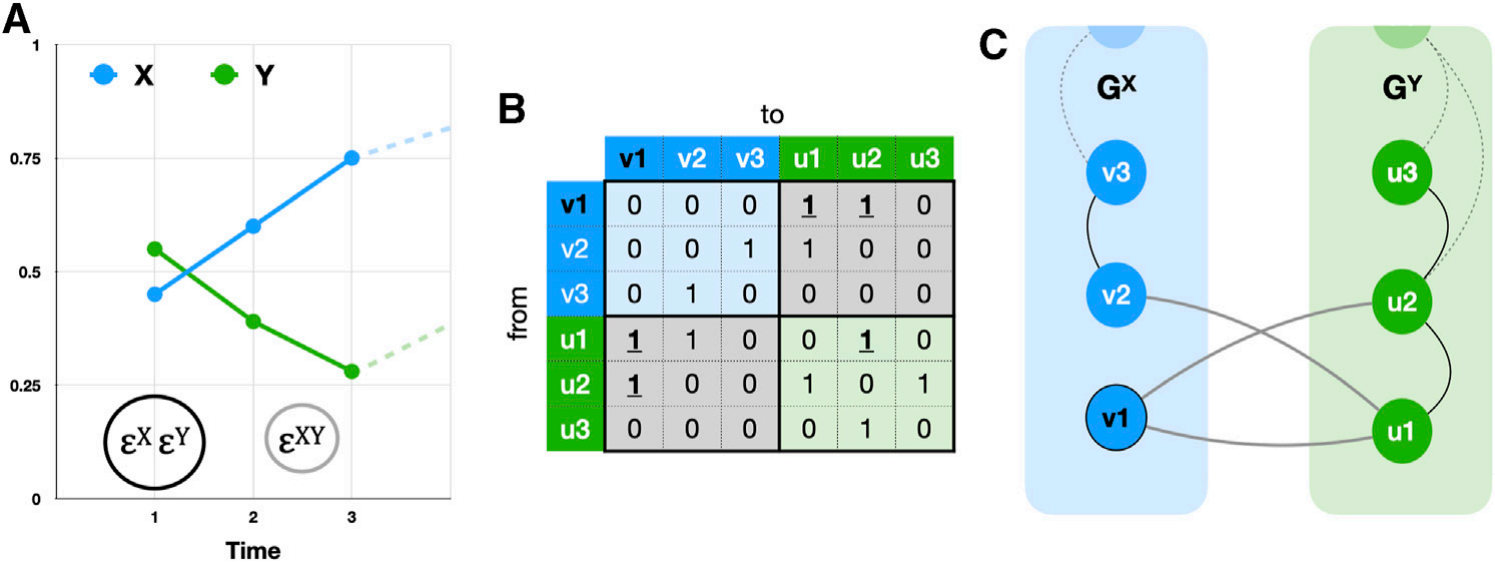
**(A)** shows two time series *X* (blue) and *Y* (green) and thresholds ε^X^ = ε^Y^ (black circle) and ε^XY^ = ε^YX^ (grey circle). **(B)** shows the composite adjacency matrix built from the recurrence matrices of X (top left, blue cells) and Y (bottom right, green cells) and their cross-recurrence matrices (bottom left and top right, grey cells). **(C)** shows two recurrence networks G^X^ and G^Y^, joined together through the cross-recurrence matrix as an interconnected recurrence network. To calculate triangle-based measures like the clustering coefficient, intersystem as well as intra system edges must be evaluated, for example, the triangle starting in **v1**: E(G^XY^) = {(v1, u1), (u1, u2), (u2, v1)}, see the bold, underlined **1**s in **(B)**. Here, the cross-edge density 𝜌^XY^ = 𝜌^YX^ = 0.33, cross-clustering *C* ^*XY*^ = 0.33 ≠ *C* ^*YX*^ = 0.

The recurrence matrix can be used to generate a graph, a so-called recurrence network (RN) (Zou et al., 2019). In a recurrence network, each vertex (node) represents a time point and each edge (a connection between 2 vertices) indicates that the values observed at these time points are recurring values, given the threshold ε . In general, to create an ε−RN of system X , the recurrence matrix $R_{ij}\left(\varepsilon \right)=R^{X}\left(\varepsilon^{X}\right)$ , with its main diagonal set to 0, is considered to be the adjacency matrix $A_{ij}\left(\varepsilon \right)=A^{X}\left(\varepsilon^{X}\right)$ of a complex network (Zou et al., 2019). This is an unweighted, undirected graph, in which each vertex $V_{i}$ represents a state of X observed at time point $t_{i}$ . For example, the graph $G^{X}$ in Figure 1C, represents the ε recurring values of time series *X* in Figure 1A. That is, the values $\left\|\vec{X_{i}}-\vec{X_{j}}\right\|$ smaller than $\varepsilon^{X}$ (the black circle in Figure 1A) are considered recurring values of *X* (Eq. 1). The top left part of the matrix in Figure 1B (blue cells) is the recurrence matrix $R^{X}\left(\varepsilon^{X}\right)$ , turned into the adjacency matrix $A^{X}\left(\varepsilon^{X}\right)$ , the rows and columns of the matrix represent the vertices of the network, a cell containing a 1 indicates the vertices are connected by an edge. In Figure 1C, vertices v2 and v3 are connected by an edge $E_{i\to j}$ with i=2 and j=2 , indicating that the state at $V_{i=2}$ recurs at $V_{j=3}$ . The construction of graph $G^{Y}$ in Figure 1C follows the same steps but is based on timeseries *Y* in Figure 1A.

### 2.2 Constructing an inter-system recurrence network

**FIGURE 2:**
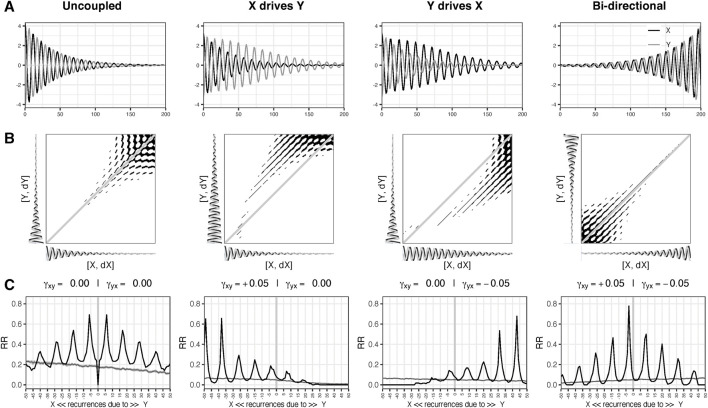
**(A)** shows simulation of 201 time steps of 2 damped oscillators X and Y (standardized) in anti-phase for 4 different types of coupling scenarios: *Uncoupled*, *X driving Y*, *Y driving X*, and *Competitive bi-directional*. The panels in row **(B)** show the Cross Recurrence Plots (CRPs) based on the joint 2D state space of *X* (X, dX) and *Y* (Y, dY). Row **(C)** shows the Diagonal Cross-Recurrence Profiles (DCRP), representing the distribution of recurrent points around the main diagonal (0 on the *x*-axis of the DCRP). The grey lines are based on 39 surrogate DCRP analyses in which the temporal order of *Y* was randomized. If the (black) line falls above or below the grey line the observed value is above/below what may be expected based on chance (*p* < .05).

An extension of RQA developed to study synchronization and coupling dynamics is called Cross-RQA (Marwan and Kurths, 2002; Shockley et al., 2002) and involves evaluating whether the state evolution of two systems X and Y can be considered close, or recurring trajectories in a shared state space. The procedure to construct a *cross-recurrence matrix* is similar to the auto-recurrence matrix, but instead of calculating the distances between the states of Y at different time points, the distances between the states of X relative to states of Y are evaluated as

$${CR}_{ij}\left(\varepsilon^{XY}\right)={CR}^{XY}\left(x_{i},y_{j}|\varepsilon^{XY}\right)=\Theta \left(\varepsilon^{XY}-\left\|\vec{x_{i}}-\vec{y_{j}}\right\|\right),\;i,j=1,\ldots ,N$$ (2)

where $\left\|∙\right\|$ is again a distance norm (e.g., Euclidean, Chebyshev) and $\Theta \left(∙\right)$ the Heaviside function. The cross-recurrence matrix is generally not symmetrical around its diagonal, in fact, the asymmetry provides information about the coupling dynamics and can be visualized using Diagonal Cross-Recurrence Profiles (DCRP) (Marwan and Kurths, 2002; Wallot and Leonardi, 2018), also known as the τ -recurrence rate (Marwan et al., 2007). Figure 2 shows Cross-Recurrence Plots (a visualization of the CR) and DCRPs for 2 coupled oscillators in 4 different coupling scenarios (see Section 3 for more details). There have been attempts to use statistical methods to study the DCRP patterns at a group level (Main et al., 2016), however, often DCRPs require visual inspection in order to make a reliable interpretation of the coupling strength and direction about individual cases. Several measures calculated from so-called inter-system recurrence networks (IRN) have been suggested that do not require such visual inspection (Donges et al., 2011; Feldhoff et al., 2012). Inter-system recurrence network can be constructed from the recurrence and cross-recurrence matrices of system X and Y . The idea is to use Cross-RQA to determine whether there should be cross-network edges, such as the grey edges between $G^{X}$ and $G^{Y}$ in Figure 1C. More formally, to study the coupling dynamics between $G^{X}$ and $G^{Y}$ we can use the cross-recurrence matrices ${CR}^{XY}\left(\varepsilon^{XY}\right)$ and ${CR}^{YX}={\left[{CR}^{XY}\left(\varepsilon^{XY}\right)\right]}^{T}$ , to build the *inter-system recurrence matrix*

$$IR\left(\varepsilon \right)=\left(\begin{array}{cc} R^{X}\left(\varepsilon^{X}\right) & {\mathrm{CR}}^{XY}\left(\varepsilon^{XY}\right)\\ {\left[{\mathrm{CR}}^{XY}\left(\varepsilon^{XY}\right)\right]}^{T} & R^{Y}\left(\varepsilon^{Y}\right) \end{array}\right)$$ (3)

with ε representing three threshold values ${\varepsilon^{X},\varepsilon^{Y},\varepsilon }^{XY}$ that can be dissimilar. It is recommended to have less cross-system recurrences than intra-system recurrences in the network, which can be controlled by choosing a threshold based on the recurrence rate (RR) it yields $\left.{RR}^{XY}\leq {\left(RR\right.}^{X}\approx {RR}^{Y}\right)$ (Feldhoff et al., 2012). $\text{IR}\left(\varepsilon \right)$ is a symmetric matrix, which can be turned into the adjacency matrix

$$A\left(\varepsilon \right)=IR\left(\varepsilon \right)-I_{N}$$ (4)

where $I_{N}$ is the identity matrix with $N=N_{X}+N_{Y}$ . This composite matrix is shown in Figure 1 Panel B. The inter-system network (IRN) is an undirected, unweighted, simple graph; however, it consists of two unipartite graphs (the recurrence networks of system X and Y ) and two bipartite graphs (the cross-recurrence networks for system X→Y and Y→X ).

### 2.3 IRN measures

The first measure of interest for an IRN is the local vertex *cross-degree*:

$$k_{v}^{XY}=\sum_{u\in V_{Y}}A_{vu}$$ (5)

with $v\in V_{X}$ , representing the number of edges that connect a vertex in $G^{X}$ to any vertex in $G^{Y}$ . This is comparable to distinguishing between the in- and out-degree in a directed network, but in the present case the purpose is to distinguish between within network and between-network connections. In Figure 1C, the cross-degrees for vertices $\left\{v_{1},v_{2},v_{3}\right\}$ of $G^{X}$ are 2,1,0, respectively, which is the same as for vertices $\left\{u_{1},u_{2},u_{3}\right\}$ of $G^{Y}$ .

#### 2.3.1 The cross-edge density

$$\rho^{XY}=\frac{1}{N_{X}N_{Y}}\sum_{v\in V_{X},u\in V_{Y}}A_{vu}$$ (6)

is a proportion representing the total number of cross-edges over the maximum possible number of edges which is equal to the cross-recurrence rate ${RR}^{XY}$ . In Figure 1C the cross-edge density is 3/9=0.33 .

The local *cross-clustering coefficient* is an estimate of the probability that two neighbors of vertex $v\in V_{X}$ randomly drawn from graph $G^{Y}$ are also neighbors

$$C_{v}^{XY}=\frac{1}{k_{v}^{YX}\left(k_{v}^{YX}-1\right)}\sum_{u_{i},u_{j}\in V_{Y}}A_{vu_{i}}A_{u_{i}u_{j}}A_{u_{j}v},\;i,j=1,\ldots ,N$$ (7)

with i≠j and for vertices with a cross-degree of 0 or 1, $C_{v}^{XY}=0$ . This clearly distinguishes the cross-clustering coefficients from Cross-RQA measures, because their calculation requires the cross-recurrence matrix as well as the auto-recurrence matrices. In Figure 1C the local clustering coefficients for $\left\{v_{1},v_{2},v_{3}\right\}$ are 1,0,0, but they are all 0 for $\left\{u_{1},u_{2},u_{3}\right\}$ . The global cross-clustering coefficient is simply the average over all vertices

$$C^{XY}={\langle C_{v}^{XY}\rangle }_{v\in V_{X}}$$ (8)

which gives $C^{XY}=0.33\neq C^{YX}=0$ . If the distribution of edges within $G^{Y}$ and between $G^{X}$ and $G^{Y}$ is uncorrelated, the expected cross-clustering coefficient would be $C^{XY}\approx \rho^{Y}\approx {RR}^{Y}$ , that is the cross-edges would be randomly and independently distributed between the graphs (Feldhoff et al., 2012). Coupling constants $C^{XY}\ll \rho^{Y}$ and $C^{XY}\gg \rho^{Y}$ indicate substantial (anti-)persistent correlations exist in the connectivity structure between the networks.

The cross-clustering coefficient measures provide information about the way the two systems are coupled and can be used to infer a coupling direction. In Section 3 we demonstrate the behavior of these measures for different coupling scenarios.

## 3 Simulation study: coupled oscillators

### 3.1 Method

The cross-clustering coefficients that can be computed from an IRN have been shown to take on different values for different coupling scenarios (Donges et al., 2011; Feldhoff et al., 2012). Predictions can be made about the expected relative magnitudes of the measures, here we demonstrate the behavior of these measures using two coupled damped oscillators, because our goal is to analyze physiological signals, which are usually periodic and may vary in amplitude and frequency over time. However, the expectations presented here do not differ from previous studies (Donges et al., 2011; Feldhoff et al., 2012).

If the oscillators are *uncoupled*, any cross-recurrences found will be spurious, however, they are expected because of the similarity between the oscillators, the only difference will be a phase lag. Therefore, the expectation is that $C^{XY}\approx C^{YX}$ . If there would be a *unidirectional coupling* X→Y of sufficient strength, it is likely that for two vertices $v_{i},v_{j}\in V_{X}$ there exists a vertex $u_{k}\in V_{Y}$ that is connected to both $v_{i}$ and $v_{j}$ . This would form a closed triplet from the perspective of $u_{k}$ , which is a vertex of the driven system Y . Therefore, the expectation would be to see $C^{YX}>C^{XY}$ for a unidirectional coupling X→Y . If the direction would be reversed and X would be the driven system Y→X , the opposite relation is expected $C^{XY}>C^{YX}$ .

The analysis scripts are available at https://osf.io/5n9kv/.

#### 3.1.1 Procedure

To study the behavior of inter-system recurrence network measures, two linear damped oscillators *X* and *Y* were simulated:

$$\frac{d^{2}X}{d^{2}t}={-\eta }_{x}X+\zeta_{x}\frac{dX}{dt}+\gamma_{yx}Y$$ (9)

$$\frac{d^{2}Y}{d^{2}t}={-\eta }_{y}Y+\zeta_{y}\frac{dY}{dt}+\gamma_{xy}X$$

Parameters $\eta_{x}$ , $\eta_{y}$ represent the frequency and $\zeta_{x}$ , $\zeta_{y}$ the damping factor of *X* and *Y* respectively. The coupling dynamics are governed by the parameters $\gamma_{xy}$ and $\gamma_{yx}$ , representing the effect of *X* on $d^{2}Y$ and *Y* on $d^{2}X$ respectively. For all simulations the initial values were $X_{0}=3$ and $Y_{0}=-3$ , resulting in an antiphase pattern. The frequency parameters were $\eta_{x}=\eta_{y}=0.3$ and the damping was set to $\zeta_{x}=\zeta_{y}=-0.05$ . The 4 coupling scenarios *Uncoupled*, *X driving Y*, *Y driving X*, and *Bi-directional (competitive)* were realized by changing the coupling parameters: $\gamma_{xy}=\gamma_{yx}$ = 0 for the *Uncoupled* scenario; $\gamma_{xy}=+0.05,\gamma_{yx}=0.00$ for the *X drives Y* scenario; $\gamma_{xy}=0.00,\gamma_{yx}=-0.05$ for the *Y drives X* scenario and $\gamma_{xy}=+0.05,\gamma_{yx}=-0.05$ for the *Bi-directional* scenario. Timeseries of length 201 were simulated using package *deSolve* (Soetaert et al., 2010) for the R computing environment (R Core Team, 2022), with default ODE solver *lsoda* (Petzold, 1983).

To analyze the data, auto-recurrence matrices for $R^{X}\left(\varepsilon^{X}\right)$ and $R^{Y}\left(\varepsilon^{Y}\right)$ and cross-recurrence matrix ${CR}^{XY}\left(\varepsilon^{XY}\right)$ were generated based on the (standardized) state vectors $\left[X,dX\right]$ and $\left[Y,dY\right]$ which describe the 2D state space of the damped oscillators (i.e., dimension = 2, but no delay embedding procedure was required to reconstruct phase space). Threshold values $\varepsilon^{X}$ and $\varepsilon^{Y}$ were chosen to yield ${RR}^{X}={RR}^{Y}=.05$ , and $\varepsilon^{XY}$ to get ${RR}^{XY}=.03$ . In addition, DCRPs were created to study the distribution of recurrent points around the main diagonal and evaluate how the interaction patterns are associated to the IRN measures.

### 3.2 Results

Figure 2 shows the cross-recurrence plots (CRP) in row B and the DCRPs in row C. Recurrent points in the diagonals of the upper triangle of the CRP are due to values occurring first in X (negative numbers on the *x*-axis of the DCRP), recurrent points in the lower triangle (positive diagonal numbers) are due to values occurring first in Y. If a peak is centered on the main diagonal this would suggest the systems are fully synchronized and display the same states at approximately the same point in time. If a peak is centered around diagonals in the lower or upper triangle of the matrix the inference can be made either system X or Y is leading the dynamics because states that occurred at an earlier point in time in X recur at a later point in time in Y , and *vice versa*. This can be seen in Figure 2, row C for the scenarios X→Y and Y→ X . If two or more off-diagonal peaks are observed, to the left and right of the main diagonal, this can point to a bi-directional coupling. In Figure 2 such a pattern can be seen for both the uncoupled and bi-directional scenarios. For the uncoupled scenario the symmetrical pattern emerges because the oscillators have the same frequency and damping parameters, there is however no peak around the main diagonal. For the bi-directional coupling there is a peak at the lag which represents the phase difference, and the pattern is symmetrical around that diagonal.

**FIGURE 3:**
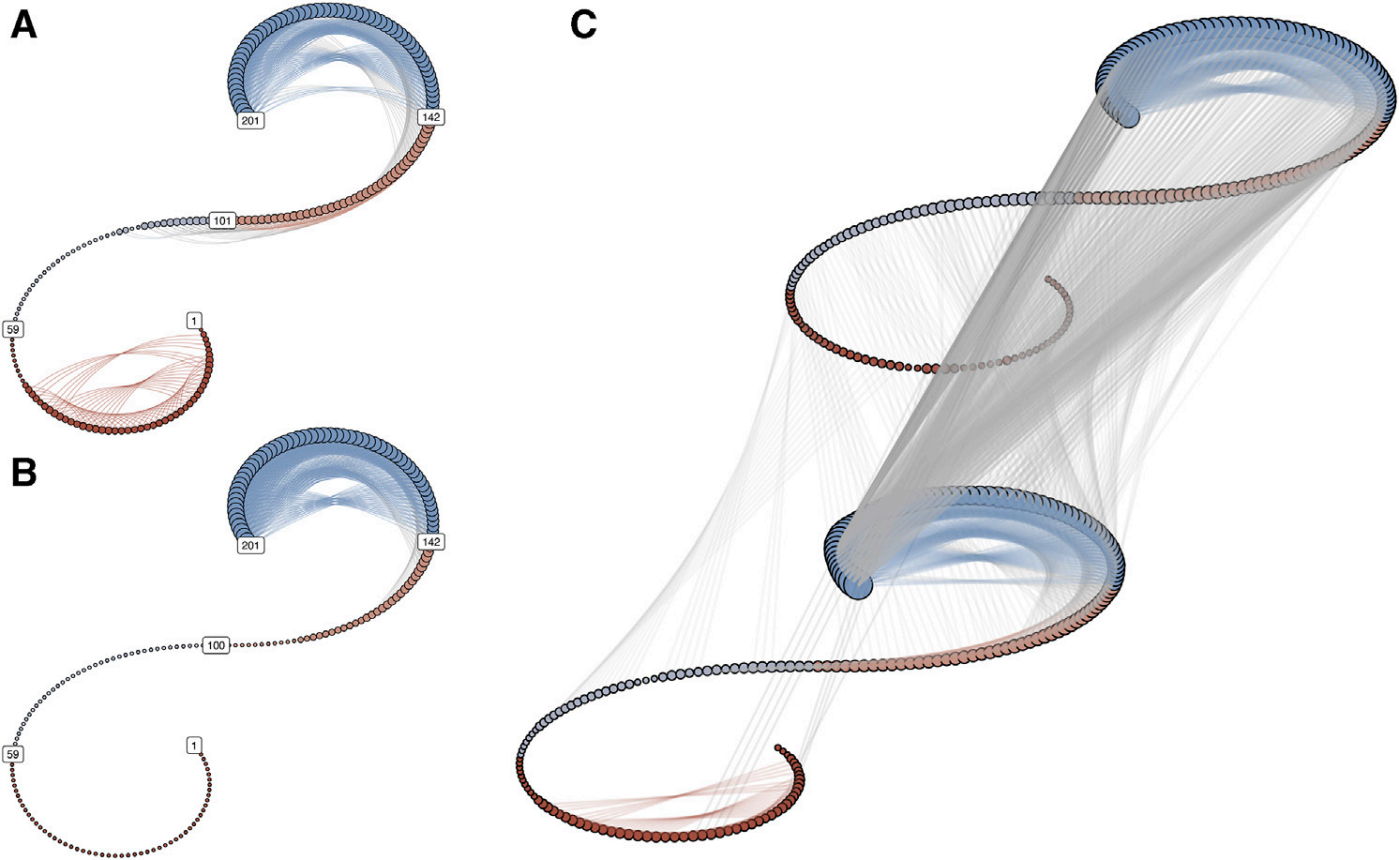
**(A,B)** represent the recurrence networks of oscillator X and Y for the coupling scenario “Y drives X.” In the spiral graph layout, the temporal order is retained, with *t* = 1 at the lower end of the spiral and *t* = 201 at the upper end. The vertices are colored to distinguish 4 epochs of equal length. If edges connect vertices from the same epoch, the edge color is the same as the vertex color. The size of the vertices scales with the vertex degree of each node. **(C)** shows the inter-system recurrence network. The grey lines connecting the spirals represent the cross-edges.

The interpretation by visual inspection of the DCRPs is facilitated by our knowledge of the system under study. With an unknown system, the unidirectional coupling would not pose a problem in this case, but distinguishing between the uncoupled scenario and the bi-directional coupling would likely be uncertain. Figure 3C shows the IRN for the scenario “Y drives X” as two coupled recurrence networks (Figures 3A, B) in a spiral layout (Hasselman and Bosman, 2020).

Table 1 shows the results for the cross-clustering coefficients, as well as the different thresholds used to generate the (cross-)recurrence matrices. The magnitude differences are all in the expected direction, that is, the difference between the uncoupled and bi-directional scenarios are ≈0 , when X→Y , $C^{YX}$ and are larger than $C^{XY}$ , which is reversed for Y→ X . These results correspond to the interpretation based on the visual inspection of the DCRPs in Figure 2. In this stylized example, the uncoupled scenario yields exactly the same cross-clustering for the different directions. This can be used to distinguish it from the bidirectional scenario, which gives a difference close to zero. This will unlikely be possible with noisy real-world empirical data. The bi-directional clustering coefficients are similar to the largest coefficients obtained for the driven scenarios, which all represent the same coupling strength magnitude in the simulation ( $\gamma_{xy}=+0.05,\gamma_{yx}=-0.05$ ).

**TABLE 1:** Inter-system recurrence network threshold parameters and clustering measures.

	ε^X^	ε^Y^	ε^XY^ = ε^YX^	C^XY^	C^YX^	ΔC
Uncoupled	.092	.092	.077	.328	.328	0
X drives Y	.092	.364	.198	.131	.388	−.257
Y drives X	.374	.092	.202	.393	.109	.284
Bi-directional	.166	.169	.120	.384	.379	.005

To study the relationship between coupling strength and the magnitude of the cross-clustering coefficient, further simulations were conducted in which $\gamma_{xy}$ and $\gamma_{yx}$ were varied from 0 to 0.1 in 40 steps. For each combination of $\gamma_{xy}$ and $\gamma_{yx}$ the difference ${∆C=C}^{XY}-C^{YX}$ was calculated, resulting in a 41 by 41 matrix of differences.

**FIGURE 4:**
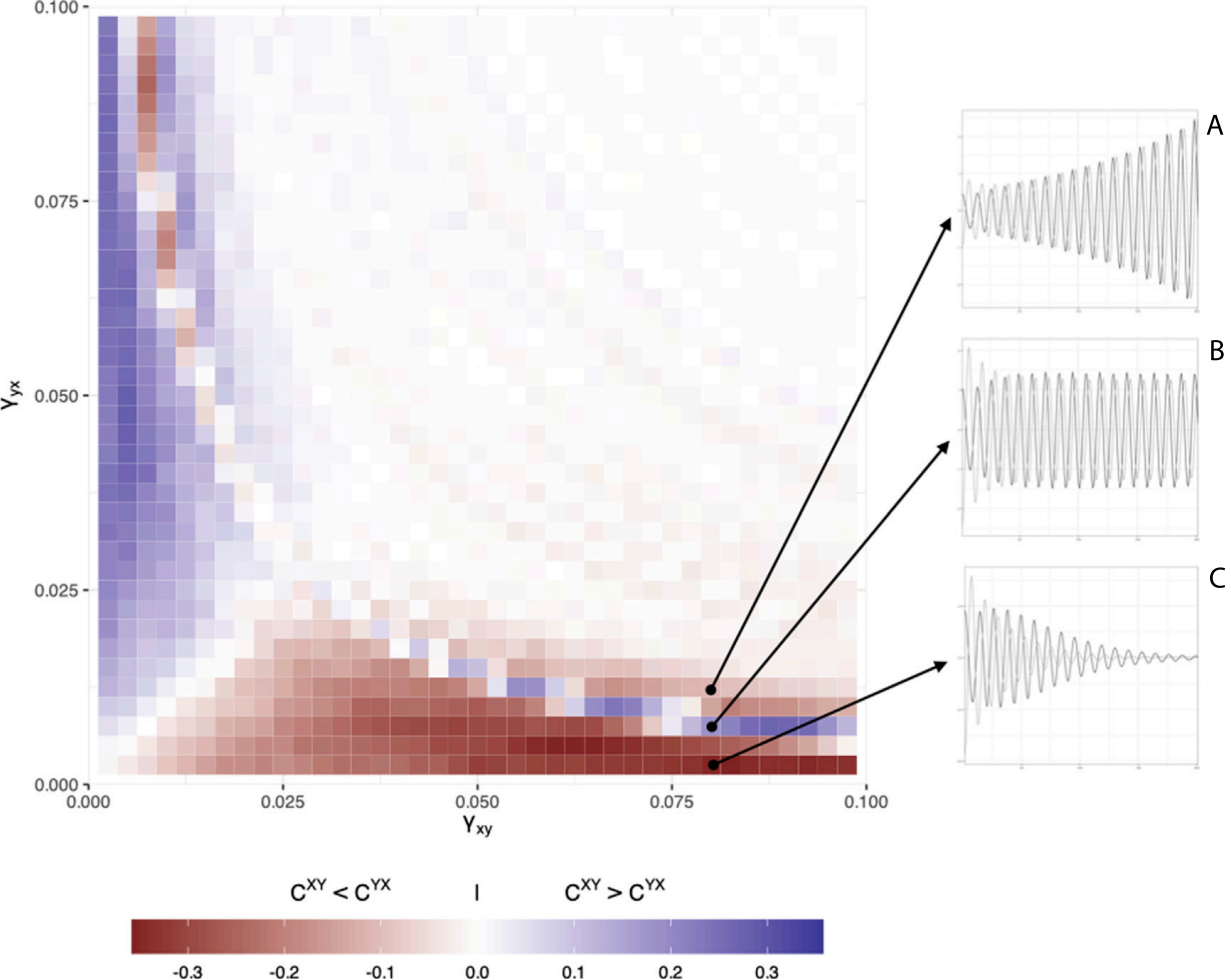
Simulation results for the 41 × 41 matrix of different coupling strength values. Color coding represents the difference ${∆C=C}^{XY}-C^{YX}$ . Red represents $C^{XY}<\;C^{YX}$ , blue $C^{XY}>\;C^{YX}$ and white $C^{XY}\approx C^{YX}$ . Three examples of oscillator dynamics are shown in the panels on the right of the matrix. The expected relationship (Y leads X) can be seen in **(A,C)**, but there is a reversal in **(B)**. See text for details.

Figure 4 is a graphical representation of the 41 by 41 matrix. It shows that if both coupling strengths become very large, the two signals become very similar and as expected, the cross-clustering coefficients do not distinguish anymore between the true coupling differences (i.e., $C^{XY}-C^{YX}\approx 0$ ). Also, there are specific combinations in which the coupling strength difference appears to indicate the opposite direction of what is expected, this occurs around the transition where the coupling strengths become stronger than the damping factor and the oscillations exponentially increase instead of decrease. The inset panels labelled A, B and C in Figure 4 show this in some more detail. Panel A and C represent the expected relationship (Y leads X) based on the coupling strengths, but the value of ∆C indicates a reversal (X leads Y) in panel B. This occurs due to the initial phase difference, when the parameter settings produce oscillator dynamics with approximately equal amplitudes that decay very slowly.

## 4 Coupling between physiological processes

### 4.1 Method

Data were collected in the context of a larger study of the relation between physiological stress and challenging behavior in clients in residential care with severe to profound intellectual disabilities (SPID) (Simons et al., 2021). The goal of the study was to examine whether information about the dynamics of physiological stress in clients with SPID and their professional caregivers can be used to understand the emergence of challenging behavior and potentially prevent incidents from occurring. In the present study we examine whether a coupling direction can be detected in physiological signals between client and caregiver in the period before an incident occurred.

#### 4.1.1 Participants, materials and procedure

The study was approved by the Faculty Ethics Committee of the Radboud University (NL-number: NL71683.091.19) 12 February 2020. Informed consent was obtained from caregivers and parents or legal representatives of clients. We analyzed 33 incidents experienced by one client-caregiver dyad over the course of 12 days between 3 August 2022 and 27 September 2022 during regular shifts of the caregiver. Both participants wore an Empatica E4 wristband (Milstein and Gordon, 2020; Schuurmans et al., 2020) which records acceleration (ACC), blood volume pressure (BVP), electrodermal activity (EDA), heart rate variability (HR) and temperature (TEMP). Whenever an incident with challenging behavior occurred the time was logged by pressing the event marker button on the wristband. After the incident, the time was also logged manually by the caregiver, providing more detailed information about the incident.

#### 4.1.2 Data analysis

**FIGURE 5:**
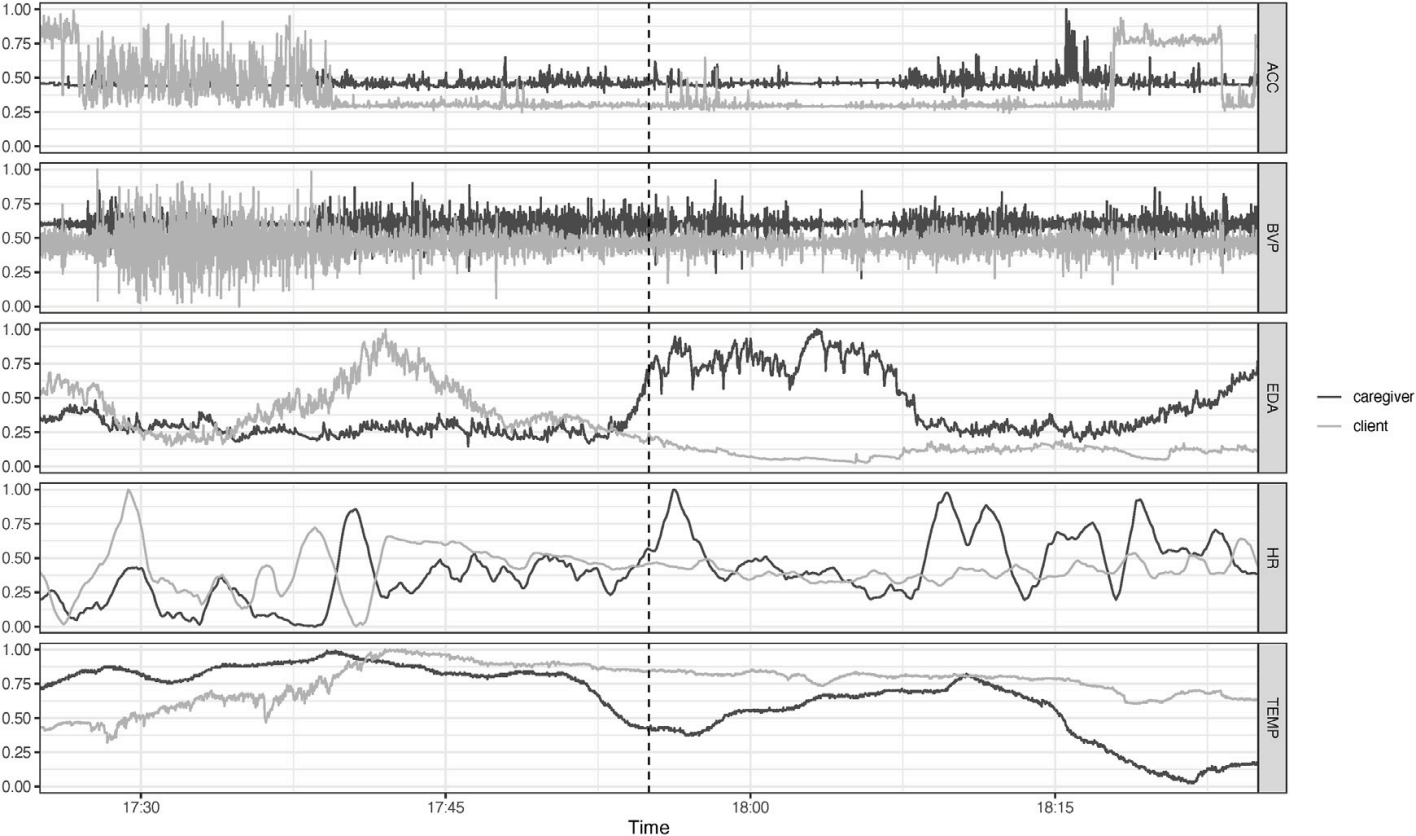
Raw time series data from caregiver and patient 30 min before and after an incident with challenging behavior occurred (dotted line at 17:55). Shown are acceleration (ACC), blood volume pressure (BVP), electrodermal activity (EDA), heart rate (HR) and temperature (TEMP).

The raw data from the Empatica E4 wristband were imported using R package *wearables* (de Looff et al., 2022). Figure 5 is an example of the data centered on the time at which an incident occurred; the analyses are based on the 35 min before the incident. There were 4 incidents for which 2 or more timeseries were shorter than 25 min. The most prominent cause was that the incident occurred within 25 min of putting on the wristband, other cases were due to sensor failure. These timeseries were removed, leaving 29 incidents with 5 timeseries. All timeseries were resampled to the same frequency (1 Hz), yielding time series of length 2,100. To construct the IRN, two auto-recurrence matrices were created (one for the client and one for the caregiver) and one cross-recurrence matrix for each incident and each variable. The matrices are based on state space vectors constructed from delay embedded time series. For each incident and variable separately, the embedding lag and embedding dimension were determined for client and caregiver using the mutual information criterion to determine the optimal embedding lag and false nearest neighbor analysis to determine the embedding dimension (Marwan et al., 2007). One set of parameters was chosen for all embeddings (embedding lag = 100 and embedding dimension = 5). The recurrence threshold for the auto-recurrence matrices was chosen to get 5% recurrent points and the cross-recurrence matrix to get 4% recurrent points. Subsequently, the local and global cross-clustering coefficients were calculated.

### 4.2 Results

**FIGURE 6:**
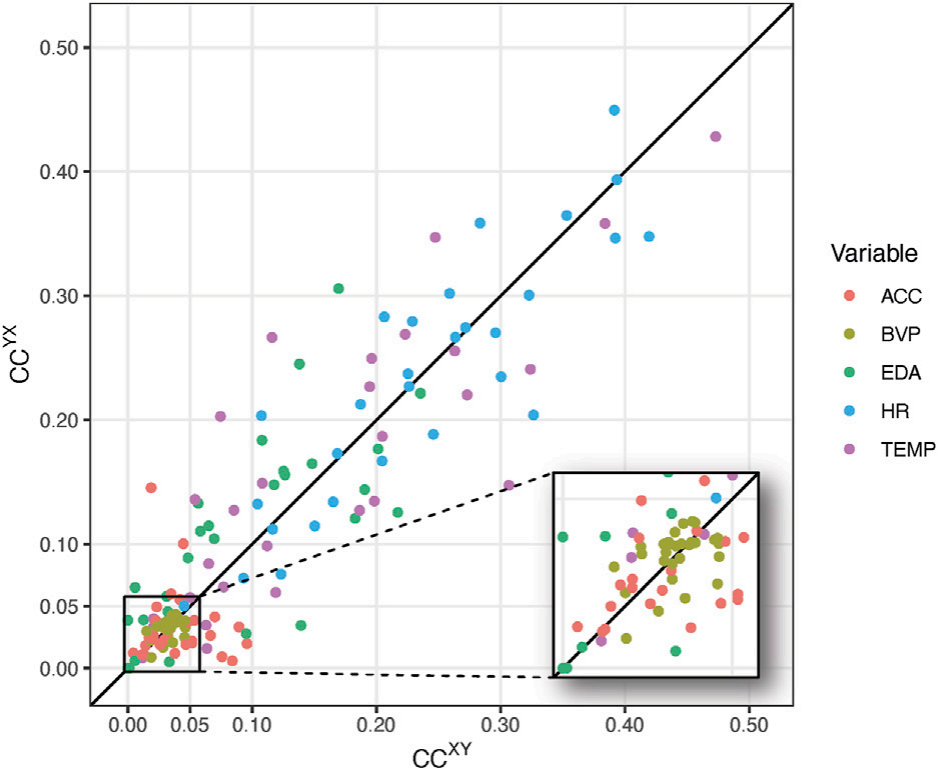
Global cross-clustering coefficients for 29 incidents for 5 different inter-system networks based on different physiological time series observed in a client (X) and a caregiver (Y). The inset shows cross-clustering coefficients in range [0,0.05].

Figure 6 displays the global cross-clustering coefficients for the 29 incidents, for the client (X) and the caregiver (Y). The location of the dots above or below the line $C^{XY}=C^{YX}$ is indicative of the coupling direction $X\to Y\;\left(C^{XY}<C^{YX}\right)$ , and $Y\to X\;\left(C^{XY}>C^{YX}\right)$ . This means the upper triangle shows cases in which the client “drives” the caregiver’s physiology, whereas the lower triangle shows cases in which the caregiver “drives” the client’s physiology. Points close to, or on the diagonal can represent uncoupled processes or bi-directional coupling dynamics.

The points in the magnified area represent cases in which $C^{XY}\ll \rho^{Y}$ and $C^{YX}\ll \rho^{X}$ , of which it is expected we should find signals with anti-persistent correlations. The results agree with these expectations as variables ACC and BVP predominantly yield values smaller than .05, whereas the coefficients for HR, EDA and TEMP are mostly larger than .05.

Table 2 shows the frequencies with which client and caregiver were driving the interaction, or whether the interaction was bi-directional or uncoupled, for each variable. If the difference $\left|C^{XY}-C^{YX}\right|\leq .01$ , this was counted as “Bi-directional/Uncoupled,” $C^{XY}-C^{YX}>.01$ as “Caregiver leading” and $C^{XY}-C^{YX}<-.01$ was counted as “Client leading.”

**TABLE 2:** Frequency of coupling directions per variable across all 29 incidents (percentage).

Variable	Client leading (%)	Caregiver leading (%)	Bi-directional uncoupled (%)
ACC	41.4	20.7	37.9
BVP	13.8	10.3	75.9
EDA	31.0	58.6	10.3
HR	41.4	34.5	24.1
TEMP	44.8	41.4	13.8
Mean	34.5	33.1	32.4

Across all 29 incidents the three types of coupling dynamics occur with equal frequency (see the row *Total* in Table 2), however, there are differences between the variables. Most notable: Compared to the caregiver the client leads twice as often for the variable ACC, for BVP the bi-directional/uncoupled type was clearly dominant, and for EDA the caregiver most often was leading the interaction.

**FIGURE 7:**
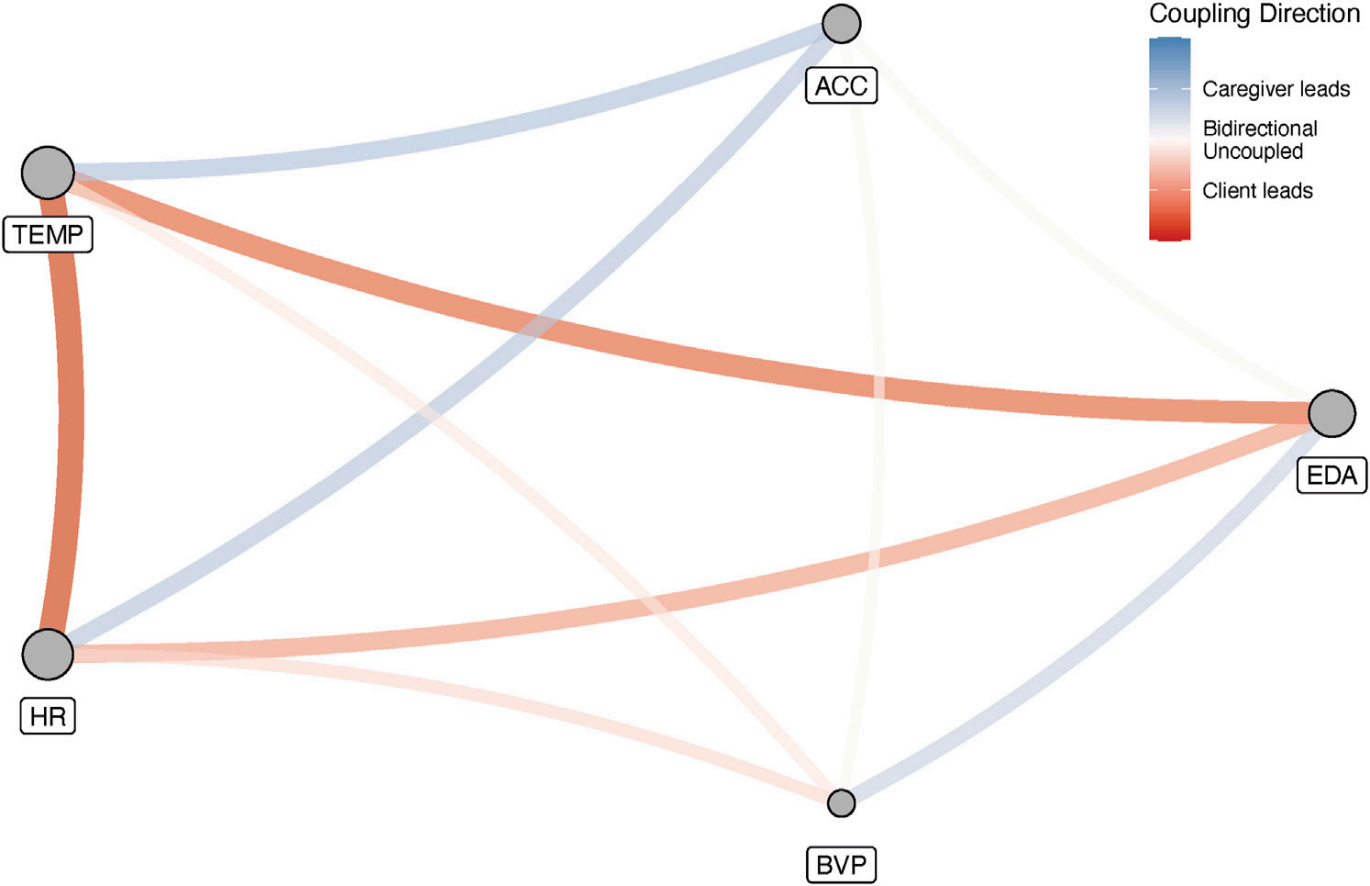
The complete multiplex inter-system recurrence network for 1 incident. Edges represent the differences between the cross-clustering coefficients for each variable. The colors are labelled according to the implied coupling direction. The vertex size reflects their strength (weighted degree).

Finally, due to the choice for one sampling frequency and one set of embedding parameters for all variables, it is possible to calculate a cross-recurrence matrix for all variable pairs, so a full IRN representing all the observed variables can be constructed. The adjacency matrix has the auto-recurrence matrices of the 5 variables on its main diagonal, all off-diagonal cells represent cross-recurrence matrices between different variable pairs. Figure 7 is a representation of such a *multiplex inter-system recurrence network* for 1 incident. The edge weights represent the absolute clustering coefficient difference and have been colored to reflect the implied coupling direction. The size of a vertex represents its weighted degree, the vertex strength (Barrat et al., 2004). For this incident an interesting path can be seen that connects the vertices with the greatest strength: The edges connecting EDA–HR–TEMP, are characterized by the client driving the dynamics. A path connecting the second largest set of 3 vertices in terms of strength, is ACC–TEMP–HR. In this loop, the dynamics between ACC–TEMP and ACC–HR are driven by the caregiver, as mentioned before, TEMP–HR is driven by the client.

## 5 General discussion and conclusion

The purpose of the present paper was to examine whether inter-system recurrence networks can be used to determine the coupling direction between physiological signals associated with stress measured simultaneously in a client-caregiver dyad, in which challenging behavior by the client can lead to incidents that are stressful for everyone involved. The question is whether such analyses can inform potential intervention or prevention efforts intended to reduce the number of incidents.

The simulation study in Section 3 provides results that are similar to previous studies (Feldhoff et al., 2012), showing that the magnitudes of the cross-coupling coefficients are associated to coupling strength and can be used to determine a coupling direction. In addition, we show that the qualification of the patterns of DCRPs agree with the inferred coupling direction. However, analogous to previous studies (Feldhoff et al., 2012), we also find parameter settings that may lead to uncertain or incorrect inferences about the coupling direction, given the true coupling strengths. In this particular case, the question is whether this should be considered an error of the method. If we were presented with the time series that yielded this result (e.g., the inset in Figure 4), absent any knowledge of the data generating process, we would conclude the estimated coupling direction to be accurate. It is indeed the case that the oscillator indicated by the cross-clustering coefficients is driving the interaction. The conclusion should be that these analyses do not recover the “true” coupling parameters, if such a thing is even possible, but instead estimate the observed, or *implied coupling direction*. An open question to be resolved is how to deal with coefficient differences close to 0. These could imply bidirectional, as well as completely uncoupled dynamics. One could argue that completely uncoupled, independent dynamics are unlikely in a measurement context in which individuals are in fact interacting, however, we cannot be completely certain based exclusively on the estimate.

The analysis of the physiological data related to 29 incidents with challenging behavior (Section 4) yielded interpretable results, as well as some corroboration of theoretical constraints on the measures described in Section 2. To start with the latter, Figure 6 clearly shows the expected relationship between (anti-)persistent correlations and edge density. The cross-clustering coefficients for the anti-persistent signal BVP are all equal or smaller than the recurrence rate of .05. For variable ACC there are 8 points outside the magnified region, but in those cases one of the two cross-clustering coefficients is always smaller than .05.

Based on Table 2 one can conclude that this dyad does not have a clear driver of the (physiological) interaction dynamics in the 35 min before an incident occurs. There are some differences between the individual variables, but Figure 7 shows that such differences at the aggregate level may be less informative to understand the interaction dynamics preceding a particular incident. The figure reveals that when all possible cross-edges between variables are considered, feedback loops can be identified that span two or more physiological signals. In Figure 7 there is clearly a strong coupling across heart rate, electrodermal activity and skin temperature that is completely driven by the client. A second motif connects heart rate and temperature to acceleration, which are both driven by the caregiver. What cannot be determined based on the current analyses is a temporal precedence for the emergence of these feedback loops. That is, we cannot claim the caregiver’s movements as measured by the acceleration variable drive the heart rate coupling dynamics which causes a reinforcing feedback loop in the client involving heartrate, electrodermal activity and temperature, that raise the stress levels of both client and caregiver to a level beyond which an incident is inevitable. The opposite scenario could also be true, in which the heart rate of the client causes the caregiver to drive the acceleration interaction dynamics, which in turn reinforces the driving of the heartrate dynamics by the client via temperature.

Future studies should explore whether imposing temporal constraints on the recurrence networks, i.e., turning them into directed networks (Hasselman, 2022) can provide more detailed information about temporal precedence in coupling dynamics. In regular directed networks, cross-clustering coefficients can be calculated to represent different types of connected triples, such as cycles, fanning in, fanning out and middleman patterns (Fagiolo, 2007). It is also possible to create weighted (and/or directed) recurrence networks (Hasselman and Bosman, 2020), for example, by keeping the distance values of the points that fall below the recurrence threshold ε in the matrix. Another option is to use recurrence times to give more weight to triangles representing states that will recur sooner rather than later in the calculation of the cross-clustering coefficient.

To summarize, the inter-system recurrence network approach to the analysis of coupled, multivariate systems produces estimates of coupling direction that are interpretable relative to the measurement context and provide insights into complex dependencies between different variables. These initial results suggest that IRN measures can at least inform *post-hoc* evaluations of interaction dynamics, which, in the present context, could occur as soon as the Empatica4 data become available. More research is required to determine whether the method can eventually be used to inform decisions related to intervention/prevention of behavior during real-time process monitoring of physiological and/or psychological variables.

## Data Availability

The raw data supporting the conclusion of this article will be made available by the authors, without undue reservation.
